# Antioxidant, hypoglycemic, and hepatoprotective effect of aqueous and ethyl acetate extract of carob honey in streptozotocin-induced diabetic rats

**DOI:** 10.14202/vetworld.2019.1916-1923

**Published:** 2019-12-10

**Authors:** Redouan El-Haskoury, Noori Al-Waili, Jaouad El-Hilaly, Waili Al-Waili, Badiaa Lyoussi

**Affiliations:** 1 Department of Biology Physiology-Pharmacology and Environmental Health Laboratory, Faculty of Sciences Dhar-Mahraz, Sidi Mohamed Ben Abdallah University, Fez, Morocco; 2 Department of Scientific Research, New York Medical Care for Nephrology, Richmond Hill, NY 11418, USA; 3 Department of Biology and Earth Sciences, Laboratory of Pedagogical Engineering and Didactics of Sciences and Mathematics (IPDSM), Regional Center for Education Careers and Training, Fez, Morocco

**Keywords:** carob honey, diabetes, ethyl acetate extract, glucose, lipid, transaminases

## Abstract

**Aim:**

The aim of the study included the effect of aqueous extract (AE) and ethyl acetate extract (EAE) on blood sugar in diabetic rats and their effects on liver enzymes and lipid panel in control and diabetic rats. Furthermore, the antioxidant activity of the EAE was studied *in vitro* and compared with AE.

**Materials and Methods:**

Sugar and antioxidant content of AE and EAE were determined. *In vitro* antioxidant activity of AE and EAE was estimated by 2, 2-diphenyl-1-picrylhydrazyl and ABTS*+ radical scavenging assay, ferric-reducing antioxidant power assay, and total antioxidant assay. To study the effect of the extracts on blood glucose level (BGL), lipid profile, and liver function in non-diabetic and diabetic rats, five groups of six rats each were treated with distilled water, AE, EAE, glibenclamide (GLB), and sucrose for 8 days. Plasma glucose level (PGL), total cholesterol (TC), triglycerides (TG), transaminases (alanine transaminase [ALT] and aspartate transaminase [AST]), and alkaline phosphatase (ALP) were determined. The effect of the interventions on BGL after acute administration also was investigated. Diabetes was induced by streptozotocin injection.

**Results:**

EAE contains significantly lower content of fructose and glucose than AE (p<0.05), and it has no sucrose. AE and EAE exhibited a significant antioxidant activity and high antioxidant content; the antioxidant content was higher in AE than EAE (p<0.05). In diabetic rats, acute treatment by AE increased PGL, while EAE significantly lowered BGL as compared to the untreated diabetic rats. Both interventions significantly decreased BGL as compared to the sucrose treated group in diabetic rats (p<0.05). EAE was more potent than GLB. Sucrose caused 13% increment in BGL after 8 days of induction of diabetes, while AE caused only 1.3% increment. Daily treatment by EAE decreased significantly AST, ALT, ALP, and TC. EAE decreased significantly TC and TG level in diabetic rats in comparison to the untreated diabetic group.

**Conclusion:**

The study showed for the 1^st^ time that EAE has more hypoglycemic effect than AE, and both extracts prevent the increment in BGL on day 8 after induction of diabetes observed in the control and sucrose treated group. EAE significantly ameliorated the lipid and liver function disorders induced by diabetes.

## Introduction

Hyperglycemia increases oxidative stress, which contributes to diabetic complications [1-3]. The Antioxidant treatment with the use of natural products could alleviate oxidative stress and complications commonly seen in diabetes [4,5]. Honey is one of the natural products that have been tested in diabetic animals and in patients with diabetes. In addition to sugars, honey contains active ingredients such as phenolics, minerals, free amino acids, enzymes, vitamins, organic acids, and other phytochemicals. Studies have shown that honey has antioxidant, anti-inflammatory, diuretic, anticancer, and antimicrobial activities [6-9]. Although honey contains a high concentration of glucose and fructose, it has important and favorable effect on blood glucose level (BGL) and lipid profile in non-diabetic and diabetic animals and in diabetic patients [10-14]. It was found that honey (1.0 or 2.0 g/kg) increases high-density lipoprotein (HDL) cholesterol while it significantly reduces hyperglycemia, triglycerides (TG), non-HDL cholesterol, coronary risk index, and cardiovascular risk index [12]. Another study showed that using honey with metformin improves glycemic control [14]. In comparison to sucrose, honey had a lower glycemic index and a peak incremental index in children with type 1 diabetes mellitus (DM) [15]. In our earlier studies, it was found that honey decreases total cholesterol (TC), low-density lipoprotein cholesterol, TG, c-reactive protein, homocysteine, and plasma glucose level (PGL), and increases HDL cholesterol in healthy individuals [11]. In diabetic patients, honey compared with dextrose and sucrose caused a significantly lower rise of PGL. Honey reduces blood lipids in normal and hyperlipidemic subjects [11]. Further results demonstrated that honey inhalation was effective in reducing BGL in healthy and diabetic subjects, and it improved glucose tolerance test and elevated plasma insulin and C-peptide [13]. Honey has been mentioned in Holy books, the Talmud, both the old and new testaments of the Bible, and the Holy Quran as a healer of diseases. In the Surat Al-Nahel (The Bee) it says (Translate the meaning): [*And your LORD taught the bee to build its cells in the mountains, on the trees and in men’s habitations, then to eat of all the fruits of the earth and find with skill the spacious paths of its LORD, their issues from within their bellies, a drink of varying colors, wherein is healing for men, verily in this is a sign for those who give thought*].

As honey contains high amount of glucose, fructose, and sucrose, its favorable effect on BGL might be mitigated by its content of carbohydrate. Therefore, the effect of ethyl acetate extract (EAE) of carob honey, which contains lower amount of sugars, on BGL in non-diabetic and diabetic rats might show better results to control high blood sugar.

The aim of the study included the effect of aqueous extract (AE) and EAE on blood sugar in diabetic rats, and their effects on liver enzymes and lipid panel in control and diabetic rats. Furthermore, the antioxidant activity of the EAE was studied *in vitro* and compared with AE.

## Materials and Methods

### Ethical approval

Ethical approval was obtained from Sidi Mohamed Ben Abdallah University Mohammed in Fez, the Animal Facility and the Laboratory of Physiology-Pharmacology and Environmental Health, Faculty of Science Dhar-Mahraz of Fez, Morocco (USMBA-PPSE 2016-05). The experiments were conducted in accordance with the accepted principles outlined in the “Guide for the Care and Use of Laboratory Animals” prepared by the National Academy of Sciences and published by the National Institutes of Health and all efforts were made to minimize animal suffering and the number of animals used.

### Carob honey aqueous and ethyl acetate extraction

Monofloral carob honey was collected in February 2016 from a beekeeper in Taounate region and was stored at room temperature for the experiment. The honey was collected from the area that was harvested by carob trees. The AE was prepared by dissolving honey in distilled water to obtain 10% (wt/v). The extract sequentially extracted 3 times with each immiscible solvent of increasing polarity using a separator funnel; the order being hexane < dichloromethane < ethyl acetate. The resultant EAE was pooled and concentrated using a rotary evaporator. The extraction yield of the EAE was approximately 10.87% of the dry weight. The extract was dissolved in water and given to the animals.

### Measurement of fructose, glucose, and sucrose in aqueous and ethyl acetate carob honey extracts

The sugar content of carob honey was determined by a method described by Liviu *et al*. [16]. Five grams of honey samples were dissolved in water and transferred to a 100 mL volumetric flask containing 25 mL of methanol; the volume was adjusted to 100 mL with water. The solution was passed through a 0.45-mm filter and the sugar content was determined in a high-performance liquid chromatography system equipped with a refractive index detector (Hitachi model L-2490, Japan). Sugar separation was performed in a Merck amino-bonded high purity silica column (LiChroCART 250-4; particle size diameter of 5 mm). The mobile phase was acetonitrile/water (75:25 v/v) at a flow rate of 1.3 mL/min and it was filtered through a membrane filter (0.45 µm) from Technochroma before the elution. The injection volume of the samples was 10 mL, with a flow rate of 1.3 mL/min. Sample peaks were identified by comparing their retention times with the standards. The samples were spiked with standards to verify the identity of the chromatographic peaks. The average peak areas of triplicate injections were used for peak quantification. A calibration curve was made for each sugar using standard solutions of different concentrations (0.5-80 mg/mL). The linear regression factor of the calibration curves was higher than 0.9982 for all sugars. The samples in crystallized form were liquefied in a 40°C water bath.

### Physicochemical parameters of carob honey

The measurements of pH, free acidity, lactone acidity, total acidity, ash, electrical conductivity, and moisture were performed according to the International Honey Commission [17]. Honey color was determined by measuring the absorbance of 50% honey solutions (wt/v) at 635 nm using a UV–visible spectrophotometer. The mm Pfund values were obtained using the following algorithm:

mm Pfund = −38.7 + 371.39 × absorbance

### Total phenolic, flavones, and flavonols determination

The total phenolic was determined by a method described by Singleton and Rossi [18]. Briefly, 100 μL of AE or EAE were mixed with 500 μL of Folin–Ciocalteu reagent solution for 6 min and then 400 μL of 20% sodium carbonate solution were added to the mixture. The absorbance was measured at 760 nm after 2 h of incubation at room temperature, and the content of total phenolics was expressed as mg of gallic acid equivalents per 100 g of the extract (mg GAE/100 g).

The content of flavones and flavonols was quantified as per follows: Briefly, 500 µl of each extract sample or standard were added to 500 µL of 2% AlCl3–ethanol solution. After 1 h at room temperature, the absorbance was measured at 420 nm. Quercetin was used as a standard, and the total content was expressed as mg of quercetin equivalents per 100 g of the extract (mg QE/100 g).

### Antioxidant activity

#### Total antioxidant activity

The total antioxidant capacity was evaluated by the phosphomolybdenum method, as described by Prieto *et al*. [19]. Briefly, 0.2 mL of AE or EAE was mixed with 2 mL of reagent solution (0.6 M sulfuric acid, 28 mM sodium phosphate, and 4 mM ammonium molybdate solutions). All the tubes were capped and incubated in a boiling water bath at 95°C for 90 min. The absorbance of the cooled mixture was measured at 695 nm, and the results were expressed as equivalents of ascorbic acid per gram of the honey extract.

#### Free radical scavenging activity: The 2, 2-diphenyl-1-picrylhydrazyl (DPPH) assay

The radical scavenging activity of the extracts against DPPH free radical was measured using the method of Clarke *et al*. [20]. Briefly, 100 µL of each extract were mixed with 825 μL of a 100 µM solution of DPPH radical prepared in ethanol (96%). The absorbance of the solution was measured at 540 nm after 15 min of incubation in the dark at room temperature. Several concentrations of samples were made, the IC_50_ (concentration of sample able to scavenge 50% of DPPH free radical) was determined.

#### ABTS*+ radical scavenging assay

The determination of ABTS*+ (2,2’-azino-bis(3-ethylbenzothiazoline-6-sulfonic acid) radical scavenging was evaluated according to the method described by Wilczyńska [21]. Briefly, 75 µL of each extract were added to 825 µL ABTS*+. The absorbance at 734 nm was measured after 6 min. Several concentrations of samples were made, the percentage inhibition was calculated, and IC_50_ (concentration of sample able to scavenge 50% of ABTS*+ free radical) was determined.

#### Ferric reducing antioxidant power (FRAP)

The FRAP of each extract was determined using the method of Saxena, Gautam, and Sharma [22]. Fifty microliter of each extract (50% w/v) in distilled water were mixed with 200 µL of 0.2 M phosphate buffer (pH 6.6) and 200 µL of 1% potassium ferricyanide. The mixture was incubated at 50°C for 20 min. Then, 200 µL of 10% trichloroacetic acid were added, and the mixture was centrifuged at 3000 rpm for 10 min. Five hundred microliter of solution from each reaction were diluted with 500 µL of distilled water, and 100 µL of 0.1% FeCl_3_ were added. The absorbance was measured at 700 nm, and ascorbic acid was used as a reference standard.

### Experimental animals

Adult male Wistar rats (230-242 g) were obtained from the animal house breeding center, Faculty of Sciences, Dhar Al-Mahraz Fez, and were housed under normal environmental conditions (25±1°C, 55±5% humidity and 12 h/12 h cycle light/dark). The animals were maintained with free access to water and standard laboratory rat’s food.

### Experimental design

Diabetes was induced by a single intravenous injection of streptozotocin (STZ) (60 mg/kg.b.wt) dissolved in a citrate buffer (0.1 M, pH 4.5). After 48 h, hyperglycemia was confirmed using a Glucometer (Accu-chek active), and animals with fasting BGL >200 mg/dl were considered diabetic and included in this study.

The hypoglycemic activity was assessed in 30 diabetic rats. After fasting for 12 h, the rats were randomly divided into five groups, six rats each.

Group1: Received distilled water and served as a control group (10 ml/kg.b.wt).

Group 2: Received AE at a dose of 1g/kg.b.wt.

Group 3: Treated with EAE at a dose of 500 mg/kg.b.wt.

Group 4: Treated with glibenclamide (GLB) at a dose of 2.5 mg/kg.b.wt.

Group 5: Received sucrose dissolve in water at a dose of 1 g/kg.b.wt.

Other 30 non-diabetic rats were assigned into five groups, six rats each, and were treated as described in the diabetic groups.

The interventions were given to the animals by gavage. Two sets of experiments were conducted; first was designed to investigate the effect of the interventions on BGL at 90 and 180 min after administration of the interventions in 16 h fasting non-diabetic and diabetic animals. The second set was designed to investigate the effect of daily ingestion of the interventions (for a total of 8 days) on BGL, alanine transaminase (ALT), aspartate transaminase (AST), alkaline phosphatase (ALP), TG, and TC. On day 8, blood was obtained from each rat by the retro-orbital puncture using capillary tubes and centrifuged at 4000 rpm for 10 min. The parameters were assessed by enzymatic methods using commercial reagent kits from SGM, Italia.

### Statistical analysis

Statistical analysis was carried out with GraphPad Prism 5.03 and R-CRAN software. Student’s t-test and one-way analysis followed by post-Tukey’s multiple comparison tests were used. Data were expressed as mean±SD; p<0.05 was considered statistically significant.

## Results

### Physicochemical parameters of carob honey and content of sugars

The data showed that the pH is 4.79±0.03, moisture is 19.5±0.4%, free acidity is 31.5±0.9 mEq/kg, lactonic acidity is 14.0±1.5 mEq/kg, total acidity is 46.0±1.9 mEq/kg, ash content is 0.65±0.01%, and electrical conductivity is 1.27±0.02 mS/cm. The honey color was amber. The EAE contains significantly (p<0.05) less amount of glucose and fructose and no sucrose as compared to the AE (Table-1).

**Table-1:** Sugar content (%) in aqueous and ethyl acetate carob honey extracts.

Honey extracts	Fructose	Glucose	Sucrose	Total sugar
Aqueous extract	40.2±0.4	33.2±0.3	0.85±0.1	73.4±0.4
Ethyl acetate extract	27.6±0.2*	19.8±0.3*	0.00	47.7±2.3*
p-value	p<0.05	p<0.05		p<0.05

* p<0.05 as compare to aqueous carob honey extract

### Total phenolic, flavones and flavonols contents, and antioxidant activity

Total phenols content of AE was 197.17±8.71 mg GAE/100 g and of EAE was 81.22±5.24 mg GAE/100 g; the difference was significant (p *<*0.05). The flavonoid content of AE was 4.39±0.38 mg QE/100 g and those of EAE extract was 3.24±0.20 mg QE/100 g; the difference was significant (p *<*0.05). Table-2 showed that AE demonstrates higher total antioxidant assay (TAA) than EAE (p *<*0.05). The TAA of AE was approximately 2.5 times higher than that of EAE extract. Both extracts decreased ABTS*+, FRAP and DPPH levels, but the effect of EAE was greater than that of AE (p *<*0.05).

**Table-2:** Antioxidant activity of aqueous and ethyl acetate extracts of carob honey.

Interventions	TAA (mg AAE/g)	ABTS (IC_50_=mg/mL)	DPPH (IC_50_=mg/mL)	FRAP (IC_50_=mg/mL)
Aqueous extract	55.25±1.10	9.17±0.25	14.34±0.17	8.62±0.18
Ethyl acetate extract	22.10±0.27*	4.17±0.15*	9.19±2.81*	5.11±0.19*
p-value	p<0.05	p<0.05	p<0.05	p<0.05

* p<0.05 as compare to aqueous extract. TAA=Total antioxidant assay, FRAP=Ferric-reducing antioxidant power, AAE=Ascorbic acid equivalent, DPPH=2, 2-Diphenyl-1-picrylhydrazyl

### Hypoglycemic effect of EAE of carob honey

In non-diabetic rats, the BGL was not significantly affected by the interventions (Table-3).

**Table-3:** Plasma glucose level after a single oral administration of carob honey aqueous extract, carob honey ethyl acetate extract, glibenclamide, and artificial honey during a period of 180 min in non-diabetic and diabetic rats.

Interventions	Non-diabetic animals				Diabetic animals			
	
	0	90 min	180 min	F/p-value	0	90	180 min	F/p-value
Water(control)	87.01±5.02	85.0±11.23	82.33±8.01	0.39/0.68	305.03±12.01	326.33±14.03*	327.5±7.52*	7.01/0.007
Honey aqueous extract	89.96±2.01	94.00±2.96	80.04±3.20*^#^	42.5/0.00	310.59±4.98	390.04±20.25*	410.12±29.06*	39.8/0.00
Honey ethyl acetate extract	88.00±7.50	90.33±4.63	89.33±6.17	6.00/0.82	290.61±29.75	186.01±23.07*	152.66±16.18*	57.22/0.00
Glibenclamide	86.66±5.78	83.33±5.04	90.31±3.67	3.17/0.07	323.03±13.21	301.00±14.11*	224.09±23.03*^#^	130/0.00
Artificial honey	88.02±3.40	91.33±7.53	89.29±7.27	0.39/0.68	309.71±6.80	420.04±16.20*	473.96±18.30*^#^	202.32/0.00

* p<0.05 as compared to 0 time.

# p<0.05 as compare to 90 min

In diabetic rats, EAE extract decreased significantly (p<0.05) BGL after 90 and 180 min of oral administration as compared to pre-treatment BGL (at 0 time) and to the control diabetic group. On the contrary, the oral administration of AE increased significantly (p<0.05) the PGL at both time intervals as compared to pre-treatment BGL and the diabetic control group. However, the elevation of BGL was significantly lower than the elevation of BGL caused by sucrose. Sucrose caused a significant elevation of BGL as compared to the AE or EAE (p<0.05). This means that both honey extracts possess a favorable effect on BGL in diabetic rats, and the effect of EAE was significantly more pronounced in the reduction of BGL. Oral administration of GLB significantly decreased PGL only after 180 min as compared to pre-treatment BGL and to the diabetic control group (p<0.05).

Daily treatment of non-diabetic rats by AE, EAE, or GLB did not significantly affect the BGL in comparison to the pre-treatment BGL and the control group (Table-4). In diabetic rats, oral administration of the EAE resulted in a significant (p<0.001) reduction of hyperglycemia as compared to the pre-treatment BGL and to the control group. Similarly, GLB significantly decreased (p<0.001) BGL on day 8 when compared to the pre-treatment BGL and to the control group.

**Table-4:** Plasma glucose changes after oral administration of aqueous extract of carob honey, ethyl acetate extract of carob honey, glibenclamide, and artificial honey during a period of 8 days in non-diabetic and diabetic rats.

Interventions	Non-diabetic animals			Diabetic animals		
	0	8	p-value	0	8	p-value
Water (control)	103.42±3.44	111.64±6.16*	0.032	349.54±32.15	380.13±30.82	0.114
Honey aqueous extract	89.69±2.01	88.56±5.66	0.660	369.34±12.15	374.37±20.83	0.626
Honey ethyl acetate extract	99.77±5.00	103.51±6.86	0.295	283.56±14.51	116.66±15.70*	<0.001
Glibenclamide	105.47±4.79	98.17±9.12	0.132	343.83±13.49	206.50±11.30*	<0.001
Artificial honey	102.42±1.74	105.40±4.50	0.691	379.34±22.15	426.37±17.32*	0.002

* p<0.05 as compared to time 0 in the same group

In the control diabetic group, BGL was increased by 9% after 8 days of the establishment of diabetes, while with the use of AE the increment was 1.3%. The use of sucrose increased BGL (13%) after 8 days as compared to the pre-treatment BGL and to the control diabetic group.

### The effect of the interventions on liver enzymes, TG, and cholesterol

In non-diabetic rats, daily treatment with AE caused an insignificant lowering of AST, TC, and TG as compared to the control group while EAE caused a significant lowering of ALP (p<0.05) (Table-5). GLB caused a significant elevation in AST and ALP levels (p<0.05) in comparison to non-diabetic control group. In diabetic rats, EAE decreased significantly (p<0.05) ALT, AST, ALP, and TC levels in comparison to the untreated diabetic group (Table-6). GLB reduced significantly ALP and TC levels (p<0.05) as compared to the diabetic control, while sucrose caused a significant elevation in AST and ALP.

**Table-5:** The effect of aqueous and ethyl acetate extracts of carob honey, glibenclamide, and artificial honey on ALT, AST, ALP, TG, and TC in nondiabetic.

Treatment groups	ALT (U/L)	AST (U/L)	ALP (U/L)	TC (mg/dL)	TG (mg/dL)
Control	37.78±3.59	72.34±3.15	80.94±6.37	85±6	196.67±16.34
Sucrose	41.55±3.28	96.12±6.22*	98.70±6.72*	103±12*	205.70±12.40
Glibencalmide	53.35±4.25*^#^	114.99±12.68*	122.37±7.62*^#^	78±1^#^	192.57±35.83
Aqueous honey extract	30.95±2.92*^#^	65.12±8.14^#^	68.52±5.29*^#^	78±5^#^	176.60±13.40
Ethyl acetate honey extract	34.18±3.91^#^	75.66±2.13^#^	46.47±5.96 *^#^	80±7^#^	191.51±18.40
F/p-value	33.7/0.00	44.9/0.00	119.4/0.00	13.14/0.00	1.45/0.24

* p<0.05 as compared to the non-diabetic group (control).

# p<0.05 as compared to the sucrose treated group. ALT=Alanine transaminase, AST=Aspartate transaminase, ALP=Alkaline phosphatase, TG=Triglycerides, TC=Total cholesterol

**Table-6:** The effect of aqueous and ethyl acetate extracts of carob honey, glibenclamide, and sucrose on ALT, AST, ALP, TG, and TC in non-diabetic and diabetic rats.

Treatment groups	ALT (U/L)	AST (U/L)	ALP (U/L)	TC (mg/dL)	TG (mg/dL)
Nondiabetic	37.78±3.59	72.34±3.15	80.94±6.37	85±6	196.67±16.34
Diabetic	81.78±1.10*	240.97±1.86*	275.18±6.23*	202±13*	242.71±38.99*
Diabetic treated by sucrose	85.55±8.82*	223.35±10.52*^π^	251.53±4.45*^π^	185±4*	251.1±8.90*
Diabetic treated by glibenclamide	71.13±4.34*	243.25±7.75*	178.47±14.11*^π#^	84±1*^π#^	183.82±34.21^π#^
Diabetic treated by aqueous honey extract	66.59±5.29*^π#^	163.25±5.25*^π#^	133.12±5.22*^π#^	145±30*	221.11±9. 45*^#^
Diabetic treated by ethyl acetate honey extract	54.59±2.82*^π#^	145.90±1.44*^π#^	103.03±4.85*^π#^	73±7*^π#^	180.37±15.81^π#^
F/p-value	80.29/0.000	816.9/0.000	669.8/0.000	97.25/0.000	10.19/0.000

* p<0.05 as compared to the non-diabetic group.

π p<0.05 as compared to the diabetic group.

# p<0.05 as compared to the sucrose treated group. ALT=Alanine transaminase, AST=Aspartate transaminase, ALP=Alkaline phosphatase, TG=Triglycerides, TC=Total cholesterol

## Discussion

The determination of physicochemical parameters helps to assess the honey quality and to identify its botanical origin. Honey physicochemical composition primarily varies with environmental conditions, harvest season, storage, the source of nectar, and the contribution of the beekeepers [22]. These factors affect the levels of honey antioxidative content, which mainly consists of phenolic acids, flavonoids, carotenoids, and enzymes [23,24]. The presented results showed that the analyzed parameters of carob honey were in accordance with the requirements of International Honey Commission 2009 and codex standard for honey [25].

The chemical analyzes of AE and EAE showed a high content of total phenolic and flavonoids in both extracts, which was significantly higher in AE. To assess the antioxidant activity of both extracts, four different methods were used: TAA, ABTS*+, FRAP, and DPPH assays. The data demonstrated that both extracts contain a considerable amount of phenolic compounds and possess significant antioxidant activity. However, AE demonstrated higher total antioxidant activity than EAE. It is not clear why EAE has lower antioxidant content as compared to AE. The method of extraction might possess an effect, which needs further investigation. It was found that aqueous, dichloromethane, and ethyl acetate fractions of acacia honey did not cause a significant antioxidant activity as compared to the pure acacia honey [26]. Therefore, fractionation of honey might decrease its antioxidant activity since its antioxidant activity is most likely due to multiple antioxidant ingredients presented in honey, which are less in fractionation parts, as evident in the present data. Therefore, fractionation of honey decreased antioxidant content and total antioxidant activity as well as sugar content. However, it increased antioxidant activity against ABTS*+, FRAP, and DPPH. It was demonstrated that antioxidant activity is attributed to the synergistic effects of different honey compounds [24,27].

Regarding the effect of the extracts on BGL, the results showed that both the acute and short-term treatment with EAE lowered BGL significantly in the diabetic rats. However, the AE raised the PGL significantly in diabetic rats, but significantly lower than the elevation of BGL caused by sucrose. In the control diabetic group, BGL was increased by 9% after 8 days of the establishment of diabetes, while with the use of AE the increment was 1.3%. It means that honey prevented further damage in the pancreases. The use of sucrose increased BGL by 13% after 8 days as compared to the pre-treatment BGL and to the control diabetic group. This shows that the use of sucrose after induction of diabetes increased significantly fasting BGL, which might indicate further damage to the pancreas. Hyperglycemia increases oxidative stress by overproduction of reactive oxygen species and by the lipid peroxidation, which can damage the pancreatic β-cell function [4,28]. The administration of AE did not reduce hyperglycemia in diabetic rats. This is most likely due to the high content of glucose and fructose. However, as compared to the sucrose, AE caused less elevation of BGL. This agrees with other studies [11,29]. Interestingly, EAE caused a significant hypoglycemic effect in diabetic rats, but had no effect on non-diabetic rats. This might be explained by the lower amount of sugars in EAE as compared to the AE. This is the first study exhibiting the beneficial effect of EAE in diabetic rats, which was more potent than AE.

Even though EAE has less total antioxidant activity than AE, its effect on BGL is more significant and will be more beneficial in diabetics. This is because high BGL stimulates free radical production; decreases the antioxidant tissue activity, and ultimately increases the insulin resistance and progression of DM [30-32]. Antioxidant activity plays a role in antidiabetic activity demonstrated by honey use. Studies have shown that flavonoids increase insulin secretion, reducing insulin resistance, and inhibit hormone-sensitive lipase activity [33-35].

ALT, AST, and ALP levels are indicators of liver function. Liver toxicity leads to an elevation of liver enzymes level in diabetic rats [36,37]. Therefore, the elevated levels of AST, ALT, and ALP in the plasma of induced diabetic rats suggest hepatocellular damage. This is obvious in the presented data that showed high level of AST, ALT, and ALP in untreated diabetic rats as compared to nondiabetic. However, treatment with EAE lowered the higher levels of AST, ALT, and ALP encountered in diabetic rats. This could be due to the hepatoprotective effect of EAE components in diabetic rats. EAE decreased TC and TG level in diabetic rats in comparison to the untreated diabetic group. Studies had reported that honey reduces cholesterol values through its antioxidant effects [38,39]. The effect of the extracts on BGL, lipid profile and liver enzymes might be more evident with the longer period of treatment.

It is known that honey has antioxidant, anti-inflammatory, and antimicrobial properties [6-9]. Moreover, it has been reported that honey has effects against hyperglycemia in diabetic patients [11,13,15]. Its anti-hyperglycemic effects may relate to its fructose content and antioxidant properties [40]. Fructose may delay the digestion and elongate the gastric emptying and decrease the absorption. The antioxidant effect of honey is due to flavonoids and phenolic acids, which may improve oxidative stress in β-cells in the pancreas, and promote insulin secretion [41,42]. In addition, honey reduces insulin resistance in type II diabetic patients [43]. However, EAE improves BGL better than whole honey in spite of lower content of fructose and antioxidant. The exact mechanism needs further investigation.

## Conclusion

This study showed that carob honey composition complies with international standards of honey. It exhibits antioxidant activity. Whole carob honey supplementation to STZ-induced diabetic rats increased BGL, which was less than BGL increment induced by sucrose administration. However, EAE of carob honey decreased BGL in diabetic rats as compared to untreated diabetic rats and the rats treated with whole carob honey. This might be due to the lower content of carbohydrates. The effect was evident despite the lower content of fructose and antioxidants than those obtained in the whole honey. EAE might be more beneficial in the management of hyperglycemia. This suggestion required further investigations, particularly with the use of interventions for a longer period.

The study showed for the first time that EAE has a more hypoglycemic effect than AE. Interestingly, both honey extracts prevent the increment in the BGL after induction of diabetes observed in the control and sucrose treated group. EAE significantly ameliorated the lipid and liver function disorders induced by diabetes. This might pave the way to investigate therapeutic effect of EAE in diabetic animals and in patient with diabetes, liver disease, and lipid disorder.

## Authors’ Contributions

RE, JE, NA, and BL designed the experimental protocols, and participated in the experimental work and writing the paper. NA analyzed the data and results and wrote the manuscript for publication. WA did the statistical analysis. All authors read and approved the final manuscript.
